# Forward-reflected-backward method with variance reduction

**DOI:** 10.1007/s10589-021-00305-3

**Published:** 2021-08-19

**Authors:** Ahmet Alacaoglu, Yura Malitsky, Volkan Cevher

**Affiliations:** 1 École Polytechnique Fédérale de Lausanne (EPFL), Lausanne, Switzerland; 2 Linköping University, Linköping, Sweden

**Keywords:** Variational inequalities, Stochastic variance reduction, Finite-sum structure, Saddle point problems, Monotone inclusions

## Abstract

We propose a variance reduced algorithm for solving monotone variational inequalities. Without assuming strong monotonicity, cocoercivity, or boundedness of the domain, we prove almost sure convergence of the iterates generated by the algorithm to a solution. In the monotone case, the ergodic average converges with the optimal *O*(1/*k*) rate of convergence. When strong monotonicity is assumed, the algorithm converges linearly, without requiring the knowledge of strong monotonicity constant. We finalize with extensions and applications of our results to monotone inclusions, a class of non-monotone variational inequalities and Bregman projections.

## Introduction

We are interested in solving variational inequalities (VI)

1 $$\begin{aligned} \text {Find } z^\star \in {\mathcal {Z}}: \langle F(z^\star ), z-z^\star \rangle + g(z) - g(z^\star ) \ge 0, \quad \forall z \in {\mathcal {Z}}, \end{aligned}$$

where *g* is a proper lower semicontinuous convex function and *F* is a monotone operator also given as the finite sum $F = \frac{1}{n} \sum _{i=1}^n F_i$.

A special case of monotone VIs is the structural saddle point problem

2 $$\begin{aligned} \min _x \max _y \Psi (x, y) + f(x) - h(y), \end{aligned}$$

where *f*, *h* are proper lower semicontinuous convex functions and $\Psi$ is a smooth convex-concave function. Indeed, problem (2) can be formulated as (1) by setting

$$\begin{aligned} z=(x,y),~~~~F(z) = \begin{bmatrix}\nabla _x\Psi (x,y) \\ -\nabla _y \Psi (x,y) \end{bmatrix},~~~~g(z) = f(x)+h(y), \end{aligned}$$

and $F(z) = \frac{1}{n}\sum _{i=1}^n F_i(z)$ (see [2, Section 2], [5, 7] for examples).

Another related problem is the monotone inclusion where the aim is to

$$\begin{aligned} \text {find}\quad z^\star \in {\mathcal {Z}}\quad \text {such that}\quad 0\in (A+F)(x), \end{aligned}$$

where $A:{\mathcal {Z}}\rightrightarrows {\mathcal {Z}}$ and $F:{\mathcal {Z}}\rightarrow {\mathcal {Z}}$ are maximally monotone operators and *F* is Lipschitz continuous with finite sum form. Monotone inclusions generalize (1) and our results also extend to this setting as will be shown in Sect. 4.1. Due to convenient abstraction, it is the problem (1) that will be our main concern.

The case when $\Psi$ in (2) is convex-concave and, in particular when it is bilinear, has found numerous applications in machine learning, image processing and operations research, resulting in efficient methods being developed in the respective areas [6, 14, 15, 33]. As VI methods solve the formulation (1), they seamlessly apply to solve instances of (2) with nonbilinear $\Psi$.

In addition to the potentially complex structure of $\Psi$, the size of the data in modern learning tasks lead to development of stochastic variants of VI methods [4, 17, 28]. An important technique on this front is stochastic variance reduction [18] which exploits the finite sum structures in problems to match the convergence rates of deterministic algorithms.

In the specific case of convex minimization, variance reduction has been transformative over the last decade [13, 16, 18, 21]. As a result, there has been several works on developing variance reduced versions of the standard VI methods, including forward-backward [2], extragradient [7, 20], and mirror-prox [5, 27]. Despite recent remarkable advances in this field, these methods rely on strong assumptions such as strong monotonicity [2, 7] or boundedness of the domain [5] and have complicated structures for handling the cases with non-bilinear $\Psi$ [5].

**Contributions** In this work, we introduce a variance reduced method with a simple single loop structure, for monotone VIs. We prove its almost sure convergence under mere monotonicity; without any of the aforementioned assumptions. The new method achieves the *O*(1/*k*) convergence rate in the general monotone case and linear rate of convergence when strong monotonicity is assumed, without using strong monotonicity constant as a parameter. We also consider natural extensions of our algorithm to monotone inclusions, a class of non-monotone problems, and monotone VIs with general Bregman distances.

**Table 1:** $^{*}$We say that the algorithm is $\mu$-adaptive if it does not require strong monotonicity constant as a parameter to obtain linear convergence. [7]) obtains $\mu$-adaptivity if cocoercivity constant of the operator is of the same order as the Lipschitz constant and not in general (see [7, Table 1]). $^\dagger$Our complexity matches the rate of deterministic methods [23, 27], however due to worse dependence on *n* compared to [5], it does not improve deterministic method in bilinear cases

	Assumptions for convergence	$\mu$-adaptivity$^{*}$	Complexity with monotonicity
[2]	Strong monotonicity	✗	N/A
[7]	Strong monotonicity	✗	N/A
[5]	Monotonicity, bounded domains	✗	${O}\left( \sqrt{n}L/\epsilon \right)$
This work	Monotonicity	✓	${O}\left( nL/\epsilon \right) ^\dagger$

### Related works

Most of the research in variance reduction has focused on convex minimization [13, 16, 18, 21], leading to efficient methods in both theory and practice. On the other hand, variance reduction for solving VIs is started to be investigated recently. One common technique for reducing the variance in stochastic VIs, is to use increasing mini-batch sizes, which leads to high per iteration costs and slower convergence rates in practice [4, 9, 17].

A different approach used in [25] was to use the same sample in both steps of stochastic extragradient method [19] to reduce the variance, which results in a slower ${O}(1/\sqrt{k})$ rate. The results of [25] for bilinear problems on the other hand are limited to the case when the matrix is full rank. The most related to our work, in the sense how variance reduction is used, are [2, 5, 7] (see Table 1).

For the specific case of strongly monotone operators, [2] proposed algorithms based on SVRG and SAGA, with linear convergence rates. Two major questions for future work are posed in [2]: *(i)* obtaining convergence without strong monotonicity assumption and *(ii)* proving linear convergence without using strong monotonicity constant in the algorithm as a parameter.

The work by [7] proposed an algorithm based on extragradient method [20] and under strong monotonicity assumption, proved linear convergence of the method. The step size in this work depends on cocoercivity constant, which might depend on strong monotonicity constant as discussed in [7, Table 1]. Thus, the result of [7] gave a partial answer to the second question of [2] while leaving the first one unanswered.

An elegant recent work of [5] focused on matrix games and proposed a method based on the mirror prox [27]. The extension of the method of [5] for general min-max problems is also considered there. Unfortunately, this extension not only features a three loop structure, but also uses the bounded domain assumption actively and requires domain diameter as a parameter in the algorithm [5, Corollary 2]. This result has been an important step towards an answer for the first question of [2].

As highlighted in Table 1, our complexity bounds have a worse dependence on *n* compared to [5], and do not improve the complexity of deterministic VI methods for bilinear games, which was the case in [5]. On the other hand, to our knowledge, our result is the first to show the existence of a variance reduced method that converges under the same set of assumptions as the deterministic methods and also matches the complexity of these deterministic methods. Moreover, our result is also the first variance reduced method to solve monotone inclusions in finite sum form, without strong monotonicity, increasing mini-batch sizes or decreasing step sizes [2].

Finally, our work answers an open problem posed in [23] regarding a stochastic extensions of the forward-reflected-backward method. Our result improves the preliminary result in [23, Section 6], which still requires evaluating the full operator every iteration.

### Preliminaries and notation

We work in Euclidean space ${\mathcal {Z}}={\mathbb {R}}^d$ with scalar product $\langle \cdot , \cdot \rangle$ and induced norm $\Vert \cdot \Vert$. Domain of a function $g:{\mathcal {Z}}\rightarrow {\mathbb {R}}\cup \{ +\infty \}$ is defined as ${{\,\mathrm{dom}\,}}g = \{ z\in {\mathcal {Z}}:g(z) < +\infty \}$. Proximal operator of *g* is defined as

$$\begin{aligned} {{\,\mathrm{prox}\,}}_{g}(u) = {{\,\mathrm{argmin}\,}}_{z\in {\mathcal {Z}}}\Bigl \{ g(z) + \frac{1}{2} \Vert z-u \Vert ^2\Bigr \}. \end{aligned}$$

We call an operator $F:{\mathcal {K}} \rightarrow {\mathcal {Z}}$, where ${\mathcal {K}}\subseteq {\mathcal {Z}}$,

- *L*-Lipschitz, for $L>0$, if   $\Vert F(u) - F(v) \Vert \le L \Vert u-v \Vert , \quad \forall u, v\in {\mathcal {K}}$.
- monotone, if   $$\langle F(u) - F(v), u - v \rangle \ge 0, \quad \forall u, v \in {\mathcal {K}}$$.
- $\nu$-cocoercive, for $\nu >0$, if  $\langle F(u) - F(v), u - v \rangle \ge \nu \Vert F(u) - F(v) \Vert ^2, \quad \forall u, v\in {\mathcal {K}}$.
- $\mu$-strongly monotone, for $\mu >0$, if  $\langle F(u) - F(v), u -v \rangle \ge \mu \Vert u-v\Vert ^2,\ \forall u,v\in {\mathcal {K}}$.

For example, in the context of (2) and (1), *F* is (strongly) monotone when $\Psi$ is (strongly) convex- (strongly) concave. However, it is worth noting that both cocoercivity and strong monotonicity fail even for the simple bilinear case when $\Psi (x,y) = \langle Ax, y\rangle$ in (2).

Given iterates $\{z_k\}_{k \ge 1}$, $\{w_k\}_{k\ge 1}$ and the filtration ${\mathcal {F}}_{k} = \sigma \{z_1, \dots , z_k, w_1, \dots , w_{k-1}\}$, we define ${\mathbb {E}}_k [\cdot ] = {\mathbb {E}} [\cdot | {\mathcal {F}}_k]$ as the conditional expectations with respect to ${\mathcal {F}}_k$.

Finally, we state our common assumptions for (1).

#### Assumption 1

- $g:{\mathcal {Z}}\rightarrow {\mathbb {R}}\cup \{+\infty \}$ is proper lower semicontinuous convex.
- $$F:{{\,\mathrm{dom}\,}}g\rightarrow {\mathcal {Z}}$$ is monotone.
- $$F =\frac{1}{n} \sum _{i=1}^nF_i$$, with *L*-Lipschitz $$F_i:{{\,\mathrm{dom}\,}}g\rightarrow {\mathcal {Z}}$$, $$\forall i$$.
- The solution set of (1), denoted by ${\mathcal {Z}}^\star$, is nonempty.

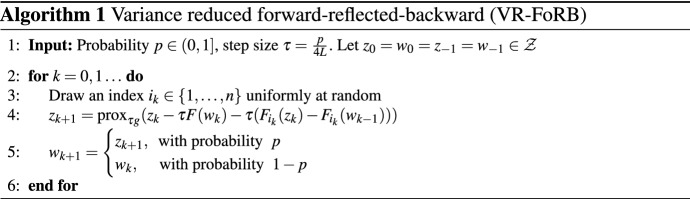


## Algorithm

Our algorithm is a careful mixture of a recent deterministic algorithm for VIs, proposed by [23], with a special technique of using variance reduction in finite sum minimization given in [16] and [21].

It is clear that for $n=1$ any stochastic variance reduced algorithm for VI reduces to some deterministic one. As a consequence, this immediately rules out the most obvious choice — the well-known *forward-backward* method (FB)

3 $$\begin{aligned} z_{k+1} = {{\,\mathrm{prox}\,}}_{\tau g}(z_k - \tau F(z_k)), \end{aligned}$$

since its convergence requires either strong monotonicity or cocoercivity of *F*. The classical algorithms that work under mere monotonicity [20, 30, 34] have a more complicated structure, and thus, it is not clear how to meld them with a variance reduction technique for finite sum problems. Instead, we chose the recent *forward-reflected-backward* method (FoRB) [23]

4 $$\begin{aligned} z_{k+1} = {{\,\mathrm{prox}\,}}_{\tau g}(z_k - \tau (2F(z_k) - F(z_{k-1}))), \end{aligned}$$

which converges under Assumption 1 with $n=1$.

When $g=0$, this method takes its origin in the Popov’s algorithm [30]. In this specific case, FoRB is also equivalent to optimistic gradient ascent algorithm [12, 31] which became increasingly popular in machine learning literature recently [11, 12, 24, 26].

Among many variance reduced methods for solving finite sum problems $\min _z f(z):=\frac{1}{n} \sum _{i=1}^n f_i(z)$ one of the simplest is the Loopless-SVRG method [21] (see also [16]),

$$\begin{aligned}&z_{k+1} = z_k - \tau \nabla f(w_k) - \tau (\nabla f_{i_k}(z_k) - \nabla f_{i_k}(w_k)) \\&w_{k+1} = {\left\{ \begin{array}{ll} z_k, \text { with probability } p, \\ w_k, \text { with probability } 1-p, \end{array}\right. } \end{aligned}$$

which can be seen as a randomized version of the gradient and hence forward-backward methods. The latter is the exact reason why we cannot extend this method directly to the variational inequality setting, without cocoercivity or strong monotonicity.

An accurate blending of [23] and [21], described above, results in Algorithm 1. Compared to Loopless-SVRG, the last evaluation of the operator at step 4 of Algorithm 1 is done at $w_{k-1}$, instead of $w_k$. In the deterministic case when $n=1$ or $p=1$, this modification reduces the method to FoRB (4) and not FB (3). The other change is that we use the most recent iterate $z_{k+1}$ in the update of $w_{k+1}$, instead of $z_k$ in the Loopless-SVRG. Surprisingly, these two small distinctions result in the method which converges for general VIs without the restrictive assumptions of the previous works.

We note that we use uniform sampling for choosing $i_k$ in Algorithm 1 for simplicity. Our arguments directly extend to arbitrary sampling as in [2, 5] which is used for obtaining tighter Lipschitz constants.

## Convergence analysis

We start with a key lemma that appeared in [23] for analyzing a general class of VI methods. The proof of this lemma is given in the appendix for completeness. The only change from [23] is that we consider the proximal operator, instead of a more general resolvent.

### Lemma 3.1

[23, Proposition 2.3] Let $g:{\mathcal {Z}}\rightarrow {\mathbb {R}}\cup \{+\infty \}$ be proper lower semicontinuous convex and let $x_1$, $U_{0}, U_1, V_1\in {\mathcal {Z}}$ be arbitrary points. Define $x_{2}$ as

5 $$\begin{aligned} x_{2} = {{\,\mathrm{prox}\,}}_g(x_1-U_1 - (V_1 - U_{0})). \end{aligned}$$

Then for all $x\in {\mathcal {Z}}$ and $V_2\in {\mathcal {Z}}$, it holds

6 $$\begin{aligned}&\Vert x_{2}-x \Vert ^2 + 2\langle V_{2}-U_1, x-x_{2}\rangle + 2\langle V_2, x_2 - x \rangle + 2g(x_2) - 2g(x) \nonumber \\&\quad \le \Vert x_{1}-x \Vert ^2 + 2\langle V_{1}-U_{0}, x-x_{1}\rangle + 2\langle V_1-U_{0},x_1-x_{2}\rangle - \Vert x_{1}-x_2 \Vert ^2. \end{aligned}$$

The benefit of Lemma 3.1 is that it gives a candidate for a Lyapunov function that can be used to prove convergence. We will need a slight modification in this function due to randomization in Algorithm 1.

### Convergence of the iterates

We start by proving the almost sure convergence of the iterates. Such a result states that the trajectories of the iterates generated by our algorithm converge to a point in the solution set. This type of result is the analogue of sequential convergence results for deterministic methods [23].

For the iterates $\{z_k\}$, $\{w_k\}$ of Algorithm 1 and any $z\in {{\,\mathrm{dom}\,}}g$, $\beta >0$ we define

$$\begin{aligned}&\Phi _{k+1}(z) :=\Vert z_{k+1} - z \Vert ^2 + 2\tau \langle F(z_{k+1}) - F(w_k), z- z_{k+1} \rangle \\&\quad + \frac{\beta }{2} \Vert z_k - w_k \Vert ^2 + \frac{1}{2} \Vert z_{k+1} - z_k \Vert ^2\\&\quad \Theta _{k+1}(z) :=\langle F(z_{k+1}), z_{k+1}-z\rangle + g(z_{k+1}) - g(z). \end{aligned}$$

The first expression plays the role of a Lyapunov function and the second is essential for the rate.

#### Lemma 3.2

Let Assumption 1 hold, $\tau < \frac{1-\sqrt{1-p}}{2L}$, $\beta = \frac{1}{\sqrt{1-p}}-1$, and the iterates $\{z_k\}$ are generated by Algorithm 1. Then for any $z\in {{\,\mathrm{dom}\,}}g$,

7 $$\begin{aligned} {\mathbb {E}}_k[ \Phi _{k+1}(z) + 2\tau \Theta _{k+1}(z)] \le \Phi _k(z). \end{aligned}$$

This lemma is essential in establishing the convergence of iterates and sublinear convergence rates that we will derive in the next section. We now continue with the proof.

#### Proof

We set in Lemma 3.1$U_0 =\tau F_i(w_{k-1})$, $U_1=\tau F(w_k)$, $V_1 =\tau F_i(z_k)$, $V_2 =\tau F(z_{k+1})$, and $x_1 = z_k$, with $i_k = i$. Then by (5) and step 4 of Algorithm 1, $x_2 = z_{k+1}$, thus, by (6)

8 $$\begin{aligned}&\Vert z_{k+1} - z \Vert ^2 + 2\tau \langle F(z_{k+1}) - F(w_k), z - z_{k+1} \rangle + 2\tau \bigl (\langle F(z_{k+1}), z_{k+1} - z \rangle \nonumber \\&\quad + g(z_{k+1}) - g(z) \bigr )\le \Vert z_k - z\Vert ^2 + 2\tau \langle F_i(z_k)- F_i(w_{k-1}), z-z_k \rangle \nonumber \\&\quad + {2\tau \langle F_i(z_k) - F_i(w_{k-1}), z_k - z_{k+1} \rangle } - \Vert z_{k+1} - z_k\Vert ^2. \end{aligned}$$

First, note that by Lipschitzness of $F_i$, Cauchy-Schwarz and Young’s inequalities,

9 $$\begin{aligned} 2\tau \langle F_i(z_k) - F_i(w_{k-1}), z_k - z_{k+1} \rangle&\le 2\tau ^2L^2 \Vert z_k - w_{k-1}\Vert ^2 + \frac{1}{2} \Vert z_k - z_{k+1} \Vert ^2. \end{aligned}$$

Thus, it follows that

10 $$\begin{aligned}&\Vert z_{k+1} - z \Vert ^2 + 2\tau \langle F(z_{k+1}) - F(w_k), z - z_{k+1} \rangle + \frac{1}{2}\Vert z_{k+1} - z_k\Vert ^2+ 2\tau \Theta _{k+1}(z)\nonumber \\&\quad \le \Vert z_k - z\Vert ^2 + 2\tau \langle F_i(z_k)- F_i(w_{k-1}), z-z_k \rangle +2\tau ^2L^2 \Vert z_k - w_{k-1}\Vert ^2. \end{aligned}$$

Taking expectation conditioning on the knowledge of $z_k, w_{k-1}$ and using that ${\mathbb {E}}_k F_i (z_k) = F(z_k)$, ${\mathbb {E}}_k F_i(w_{k-1}) = F(w_{k-1})$, we obtain

11 $$\begin{aligned}&{\mathbb {E}}_k \Vert z_{k+1} - z \Vert ^2 + 2\tau {\mathbb {E}}_k \langle F(z_{k+1}) - F(w_k), z - z_{k+1} \rangle +\frac{1}{2} {\mathbb {E}}_k \Vert z_{k+1} -z_k\Vert ^2 \nonumber \\&\quad +2\tau {\mathbb {E}}_k \Theta _{k+1}(z) \le \Vert z_k - z\Vert ^2 + 2\tau \langle F(z_k) - F(w_{k-1}), z-z_k \rangle \nonumber \\&\qquad\qquad\qquad\qquad + 2\tau ^2L^2 \Vert z_{k} - w_{k-1} \Vert ^2. \end{aligned}$$

Adding

12 $$\begin{aligned} \frac{\beta }{2}{\mathbb {E}}_{k} \Vert z_k - w_k \Vert ^2 = \frac{\beta (1-p)}{2} \Vert z_k - w_{k-1} \Vert ^2, \end{aligned}$$

which follows from the definition of $w_k$, to (11), we obtain

13 $$\begin{aligned}&{\mathbb {E}}_k [\Phi _{k+1}(z) +2\tau \Theta _{k+1}(z)] \le \Phi _{k}(z) \nonumber \\&\quad +\Big (2\tau ^2L^2+\frac{\beta (1-p)}{2} \Big )\Vert z_{k} - w_{k-1} \Vert ^2-\frac{1}{2}\Vert z_k-z_{k-1} \Vert ^2 \nonumber \\&\quad -\frac{\beta }{2}\Vert z_{k-1}-w_{k-1} \Vert ^2. \end{aligned}$$

The proof will be complete, if we can show that the expression in the second and third lines are nonpositive. Due to our choice of $\beta$ and $\tau$ this is a matter of a simple algebra. As $\beta + 1 = \frac{1}{\sqrt{1-p}}$, $\frac{\beta }{1+\beta } = 1-\sqrt{1-p}$, and $2\tau L < 1 - \sqrt{1-p} = \frac{\beta }{1+\beta }$, we have

14 $$\begin{aligned} 2\tau ^2L^2+\frac{\beta (1-p)}{2}\le \frac{1}{2}\Bigl (\frac{\beta ^2}{(1+\beta )^2} + \frac{\beta }{(1+\beta )^2}\Bigr ) = \frac{\beta }{2(1+\beta )}. \end{aligned}$$

Then we must show that

$$\begin{aligned} \frac{\beta }{1+\beta }\Vert z_k-w_{k-1} \Vert ^2\le \Vert z_k-z_{k-1} \Vert ^2 + \beta \Vert z_{k-1}-w_{k-1} \Vert ^2, \end{aligned}$$

which is a direct consequence of $\Vert u+v \Vert ^2 \le (1+\frac{1}{\beta })\Vert u \Vert ^2 + (1+\beta )\Vert v \Vert ^2$. The proof is complete. $\square$

#### Theorem 3.1

Let Assumption 1 hold and let $\tau < \frac{1-\sqrt{1-p}}{2L}$. Then for the iterates $\{z_k\}$ of Algorithm 1, almost surely there exists $z^\star \in {\mathcal {Z}}^\star$ such that $z_k \rightarrow z^\star$.

#### Remark 3.1

It is interesting to observe that for $p=1$, i.e., when the algorithm becomes deterministic, the bound for the stepsize is $\tau < \frac{1}{2L}$, which coincides with the one in [23] and is known to be tight. In this case analysis will be still valid if for convenience we assume that $\infty \cdot 0 = 0$.

For small *p* we might use a simpler bound for the stepsize, as the following corollary suggests.

#### Corollary 3.1

Suppose that $p=\frac{1}{n}$ and $\tau \le \frac{p}{4L}=\frac{1}{4Ln}$. Then the statement of Theorem 3.1 holds.

#### Proof

We only have to check that $\frac{p}{2}\le 1 - \sqrt{1-p}$, which follows from $\sqrt{1-p}\le 1 - \frac{p}{2}$.$\square$

#### Proof of Theorem 3.1

From Lemma 3.2 we have for any $z\in {{\,\mathrm{dom}\,}}g$

$$\begin{aligned} {\mathbb {E}}_{k} [\Phi _{k+1}(z) +2\tau \Theta _{k+1}(z)] \le \Phi _{k}(z). \end{aligned}$$

First, we show that $\Phi _{k+1}(z)$ is nonnegative for all $z\in {{\,\mathrm{dom}\,}}g$. This is straightforward but tedious. Recall that $1-\sqrt{1-p} = \frac{\beta }{1+\beta }$ and hence $2\tau L\le \frac{\beta }{1+\beta }$. Then by Cauchy-Schwarz and Young’s inequalities,

15 $$\begin{aligned} -2\tau \langle&F(z_{k+1}) - F(w_k),z- z_{k+1} \rangle \le 2\tau L\Vert z_{k+1}-w_k \Vert \Vert z_{k+1}-z \Vert \nonumber \\&\le \frac{\beta }{2(1+\beta )}\Big (\Vert z_{k+1}-w_k \Vert ^2+\Vert z_{k+1}-z \Vert ^2\Big )\nonumber \\&\le \frac{\beta }{2(1+\beta )}\Vert z_{k+1}-z \Vert ^2 + \frac{\beta }{2(1+\beta )}\Big (\big (1+\frac{1}{\beta }\big )\Vert z_{k+1}-z_k \Vert ^2 \nonumber \\&\qquad\qquad\qquad\qquad\qquad\qquad + (1+\beta )\Vert z_k-w_k \Vert ^2\Big )\nonumber \\&= \frac{\beta }{2(1+\beta )}\Vert z_{k+1}-z \Vert ^2 + \frac{1}{2}\Vert z_{k+1}-z_k \Vert ^2 +\frac{\beta }{2}\Vert z_k-w_k \Vert ^2. \end{aligned}$$

Therefore, we deduce

16 $$\begin{aligned} \Phi _{k+1}(z) \ge \Vert z_{k+1} - z\Vert ^2 - \frac{\beta }{2(1+\beta )}\Vert z_{k+1}-z \Vert ^2 \ge \frac{1}{2} \Vert z_{k+1}-z \Vert ^2. \end{aligned}$$

Now let $z={\bar{z}} \in {\mathcal {Z}}^{\star }$. Then by monotonicity of *F* and (1),

17 $$\begin{aligned}&\Theta _{k+1}({\bar{z}})= \langle F(z_{k+1}), z_{k+1} - {\bar{z}} \rangle +g(z_{k+1}) - g({\bar{z}}) \nonumber \\&\quad \ge \langle F({\bar{z}}), z_{k+1} - {\bar{z}} \rangle +g(z_{k+1}) - g({\bar{z}})\ge 0. \end{aligned}$$

Summing up, we have that $\Theta _{k+1}({\bar{z}}) \ge 0$, $\Phi _k({\bar{z}})\ge 0$ and ${\mathbb {E}}_{k}\Phi _{k+1}({\bar{z}})\le \Phi _k({\bar{z}})$. Unfortunately, this is still not sufficient for us, so we are going to strengthen this inequality by reexamining the proof of Lemma 3.2. In estimating the second line of inequality (13) we used that $2\tau L\le 1 - \sqrt{1-p}$, however, both in the statements of Lemma 3.2 and Theorem 3.1 we assumed a strict inequality. Let

18 $$\begin{aligned}&\delta = \frac{\beta }{1+\beta }-\frac{4\tau ^2 L^2(1+\beta )}{\beta } \nonumber \\& \iff 4\tau ^2L^2 = \frac{\beta ^2}{(1+\beta )^2}-\frac{\delta \beta }{1+\beta}. \end{aligned}$$

From $2\tau L < 1 - \sqrt{1-p} = \frac{\beta }{1+\beta }$ it follows that $\delta >0$. Now, inequality (14) can be improved to equality as

19 $$\begin{aligned} 2\tau ^2L^2+\frac{\beta (1-p)}{2} = \frac{1}{2}\Bigl (\frac{\beta ^2}{(1+\beta )^2} -\frac{\delta \beta }{(1+\beta )}+ \frac{\beta }{(1+\beta )^2}\Bigr ) = \frac{\beta (1-\delta )}{2(1+\beta )}. \end{aligned}$$

This change results in a slightly stronger version of (7)

20 $$\begin{aligned}&{\mathbb {E}}_k [\Phi _{k+1}({\bar{z}}) +2\tau \Theta _{k+1}({\bar{z}})]\nonumber \\&\quad \le \Phi _{k}({\bar{z}}) -\frac{\delta }{2}\Bigl (\Vert z_k-z_{k-1} \Vert ^2 + \beta \Vert z_{k-1}-w_{k-1} \Vert ^2\Bigr ). \end{aligned}$$

As $\Phi _{k+1}({\bar{z}})\ge 0$ and $\Theta _{k+1}({\bar{z}})\ge 0$, we can apply Robbins-Siegmund lemma [32] to conclude that $\{\Phi _{k+1}({\bar{z}})\}$ converges almost surely and that

21 $$\begin{aligned} \sum _{k=1}^\infty {\mathbb {E}} \left[ \Vert z_k - z_{k-1} \Vert ^2 + \Vert z_{k-1} - w_{k-1} \Vert ^2\right] < \infty . \end{aligned}$$

It then follows that almost surely, $\Vert z_k - z_{k-1} \Vert ^2 \rightarrow 0$ and $\Vert z_{k-1} - w_{k-1} \Vert ^2 \rightarrow 0$. Moreover, due to (16), $\{ z_k \}$ is almost surely bounded and therefore by the definition of $\Phi _k$, continuity of *F*, and (21), we have that $\Vert z_k - {\bar{z}}\Vert ^2$ converges almost surely.

More specifically, this means that for every ${\bar{z}}\in {\mathcal {Z}}^\star$, there exists $\Omega _{{\bar{z}}}$ with ${\mathbb {P}}(\Omega _{{\bar{z}}}) = 1$ such that $\forall \omega \in \Omega _{{\bar{z}}}$, $\Vert z_k(\omega ) - {\bar{z}} \Vert ^2$ converges. We can strengthen this result by using the arguments from [3, Proposition 9], [8, Proposition 2.3] to obtain that there exists $\Omega$ with ${\mathbb {P}}(\Omega )=1$ such that for every ${\bar{z}}\in {\mathcal {Z}}^\star$ and for every $\omega \in \Omega$, $\Vert z_k(\omega ) -{\bar{z}} \Vert ^2$ converges.

We now pick a realization $\omega \in \Omega$ and note that $z_k(\omega ) - z_{k-1}(\omega ) \rightarrow 0$ and $z_{k-1}(\omega ) - w_{k-1}(\omega ) \rightarrow 0$. Let us denote by ${\tilde{z}}$ a cluster point of the bounded sequence $z_k(\omega )$. By using the definition of $z_{k}$ and convexity of *g*, as in the proof of Lemma 3.1, we have for any $z \in {\mathcal {Z}}$

$$\begin{aligned} g(z) \ge g(z_{k}(\omega ))&+ \frac{1}{\tau }\langle z_{k-1}(\omega ) -z_{k}(\omega ),z-z_k(\omega )\rangle - \langle F(w_{k-1}(\omega )), z-z_k(\omega )\rangle \\&- \langle F_{i_{k-1}}(z_{k-1}(\omega )) - F_{i_{k-1}}(w_{k-2}(\omega )), z - z_{k}(\omega )\rangle . \end{aligned}$$

Taking the limit as $k\rightarrow \infty$ and using that *g* is lower semicontinuous and $\forall i$, $F_{i}$ is Lipschitz, $z_k(\omega ) - z_{k-1}(\omega ) \rightarrow 0$ and $z_{k-1}(\omega ) - w_{k-1}(\omega ) \rightarrow 0$, we get that ${\tilde{z}}\in {\mathcal {Z}}^\star$. Then, as we have that $\Vert z_{k}(\omega ) - {\tilde{z}} \Vert ^2$ converges and we have shown that $\Vert z_k(\omega ) - {\tilde{z}} \Vert ^2$ converges to 0 at least on one subsequence, we conclude that the sequence $(z_k(\omega ))$ converges to some point ${\tilde{z}}$, where ${\tilde{z}}\in {\mathcal {Z}}^\star$.$\square$

### Convergence rate for the general case

In this section, we prove that the average of the iterates of the algorithm exhibits *O*(1/*k*) convergence rate which is optimal for solving monotone VIs [27]. The standard quantity to show sublinear rates for VIs is gap function which is defined as

$$\begin{aligned} G({\bar{z}}) = \sup _{z\in {\mathcal {Z}}} \langle F(z), {\bar{z}}-z \rangle + g({\bar{z}}) - g(z). \end{aligned}$$

As this quantity requires taking a supremum over the whole space ${\mathcal {Z}}$ which is potentially unbounded, restricted versions of gap functions are used, for example in [22, 29]

22 $$\begin{aligned} G_{{\mathcal {C}}}({\bar{z}}) = \sup _{z\in {\mathcal {C}}} \langle F(z), {\bar{z}}- z \rangle + g({\bar{z}}) - g(z), \end{aligned}$$

where ${\mathcal {C}} \subset {{\,\mathrm{dom}\,}}g$ is an arbitrary bounded set. It is known that $G_{{\mathcal {C}}}({\bar{z}})$ is a valid merit function, as proven by [29, Lemma 1]. As we are concerned with randomized algorithms, we derive the rate of convergence for the expected gap function ${\mathbb {E}}\left[ G_{{\mathcal {C}}}(z_k)\right]$.

#### Theorem 3.2

Given $\{z_k\}$ generated by Algorithm 1, we define the averaged iterate $z_K^{av} = \frac{1}{K} \sum _{k=1}^K z_k$. Let ${\mathcal {C}} \subset {{\,\mathrm{dom}\,}}g$ be an arbitrary bounded set. Then under the hypotheses of Theorem 3.1 it holds that

$$\begin{aligned} {\mathbb {E}} \left[ G_{{\mathcal {C}}}(z_K^{av})\right] \le \frac{1}{K} \left[ \frac{1}{\tau }\sup _{z\in {\mathcal {C}}} \Vert z_0 - z \Vert ^2 + \frac{2\tau L^2(1+\beta )}{\delta \beta }{{\,\mathrm{dist}\,}}(z_0,{\mathcal {Z}}^\star )^2\right] , \end{aligned}$$

where $$\delta = \frac{\beta }{1+\beta }-\frac{4\tau ^2 L^2(1+\beta )}{\beta }$$.

#### Remark 3.2

If we set $p=\frac{1}{n}$, $\tau =\frac{p}{3\sqrt{2} L}$, and $\beta = \frac{1}{\sqrt{1-p}}-1$, the rate will be bounded by $\frac{nL}{K}\left( 3\sqrt{2} \sup _{z\in {\mathcal {C}}}\Vert z_0-z \Vert ^2+12\sqrt{2} {{\,\mathrm{dist}\,}}(z_0,{\mathcal {Z}}^\star )^2\right)$, hence it is ${O}(\frac{nL}{K})$.

The high level idea of the proof is that on top of Lemma 3.2 we sum the resulting inequality and accumulate terms $\Theta _{k}(z)$. Then we use Jensen’s inequality to obtain the result.

There are two intricate points that need attention in these kind of results. First, the convergence measure is the expected duality gap ${\mathbb {E}}[G_{{\mathcal {C}}}(z_K^{av})]$ that includes the expectation of the supremum. In a standard analysis, it is easy to obtain a bound for the supremum of expectation, however obtaining the former requires a technique, which is common in the literature for saddle point problems [1, 28]. Roughly, the idea is to use an auxiliary iterate to characterize the difference two quantities, and show that the error term does not degrade the rate.

Second, as duality gap requires taking a supremum over the domain, the rate might contain a diameter term as in [5]. The standard way to adjust this result for unbounded domains is to utilize a restricted merit function as in (22) on which the rate is obtained [29]. We note that the result in [5] not only involves the domain diameter in the final bound, but it also requires the domain diameter as a parameter for the algorithm in the general monotone case [5, Corollary 2].

#### Proof of Theorem 3.2

First, we collect some useful bounds. Consider (20) with a specific choice ${\bar{z}} = P_{{\mathcal {Z}}^\star }(z_0)$. Taking a full expectation and then summing that inequality, we get

23 $$\begin{aligned}&\frac{\delta }{2}\sum _{k=0}^{\infty } {\mathbb {E}}\left[ \Vert z_k - z_{k-1} \Vert ^2 + \beta \Vert z_{k-1} - w_{k-1} \Vert ^2 \right] \nonumber \\&\quad \le \Vert z_0 - P_{{\mathcal {Z}}^\star }(z_0) \Vert ^2 ={{\,\mathrm{dist}\,}}(z_0, {\mathcal {Z}}^*)^2, \end{aligned}$$

which also implies by Young’s inequality that

24 $$\begin{aligned} \frac{\beta \delta }{2(1+\beta )} \sum _{k=0}^{\infty } {\mathbb {E}}\Vert z_k - w_{k-1}\Vert ^2 \le {{\,\mathrm{dist}\,}}(z_0, {\mathcal {Z}}^*)^2. \end{aligned}$$

Next, we rewrite (10) as

25 $$\begin{aligned}&2\tau \Theta _{k+1}(z) + \Vert z_{k+1} - z \Vert ^2 + 2\tau \langle F(z_{k+1}) - F(w_k), z - z_{k+1} \rangle + \frac{1}{2}\Vert z_{k+1} - z_k\Vert ^2 \nonumber \\&\quad \le \Vert z_k - z\Vert ^2 + 2\tau \langle F(z_k)- F(w_{k-1}), z-z_k \rangle +2\tau ^2L^2 \Vert z_k - w_{k-1}\Vert ^2\nonumber \\&\quad + 2\tau \langle F_{i_k}(z_k)- F_{i_k}(w_{k-1}) - (F(z_k) - F(w_{k-1})), z-z_k \rangle . \end{aligned}$$

Let $\nu _k = \tau (F_{i_k}(z_k)- F_{i_k}(w_{k-1}) - (F(z_k) - F(w_{k-1})))$, then ${\mathbb {E}}_k \left[ \nu _k \right] = 0$. We define the process $\{{\hat{z}}_k\}$ by ${\hat{z}}_0 = z_0$ and

26 $$\begin{aligned} {\hat{z}}_{k+1} = {\hat{z}}_{k} + \nu _k. \end{aligned}$$

Note that for ${\mathcal {F}}_k = \sigma \{z_1,\dots , z_k,w_1,\dots , w_{k-1}\}$, ${\hat{z}}_k$ is ${\mathcal {F}}_k$-measurable. It also follows that $\forall z\in {\mathcal {Z}}$

27 $$\begin{aligned} \Vert {\hat{z}}_{k+1} - z\Vert ^2 = \Vert {\hat{z}}_k - z \Vert ^2 + 2\langle \nu _k, {\hat{z}}_k - z \rangle + \Vert \nu _k \Vert ^2, \end{aligned}$$

which after summation over $k=0,\dots , K-1$ yields

28 $$\begin{aligned} \sum _{k=0}^{K-1} 2\langle \nu _k, z - {\hat{z}}_k \rangle \le \Vert z_0 - z\Vert ^2 + \sum _{k=0}^{K-1} \Vert \nu _k \Vert ^2. \end{aligned}$$

With the definition of $\nu _k$ we can rewrite (25) as

$$\begin{aligned}&2\tau \Theta _{k+1}(z) + \Vert z_{k+1} - z \Vert ^2 + 2\tau \langle F(z_{k+1}) - F(w_k), z - z_{k+1} \rangle + \frac{1}{2}\Vert z_{k+1} - z_k\Vert ^2\\&\quad \le \Vert z_k - z\Vert ^2 + 2\tau \langle F(z_k)- F(w_{k-1}), z-z_k \rangle +2\tau ^2L^2 \Vert z_k - w_{k-1}\Vert ^2\\&\quad + 2\langle \nu _k , z- {\hat{z}}_k \rangle + 2\langle \nu _k , {\hat{z}}_k - z_k \rangle . \end{aligned}$$

We use (12), the definition of $\Phi _k$, and the arguments in Lemma 3.2 to show that the last line of (13) is nonpositive, to obtain

29 $$\begin{aligned}&2\tau \Theta _{k+1}(z) + \Phi _{k+1}(z) +\frac{\beta }{2}\Bigl ( {\mathbb {E}}_{k}\Vert z_k-w_k \Vert ^2-\Vert z_k-w_k \Vert ^2\Bigr )\nonumber \\&\quad \le \Phi _k(z) + 2\langle \nu _k , z- {\hat{z}}_k \rangle + 2\langle \nu _k , {\hat{z}}_k - z_k \rangle . \end{aligned}$$

Summing this inequality over $k=0,\dots , K-1$ and using bound (28) yields

30 $$\begin{aligned}&2\tau \sum _{k=0}^{K-1} \Theta _{k+1}(z) + \Phi _{K}(z) + \frac{\beta }{2}\sum _{k=0}^{K-1}\Bigl ({\mathbb {E}}_{k}\Vert z_k-w_k \Vert ^2-\Vert z_k-w_k \Vert ^2\Bigr )\nonumber \\&\le \Phi _0(z) +2\sum _{k=0}^{K-1}\langle \nu _k , z- \hat{z}_k \rangle + 2\sum _{k=0}^{K-1}\langle \nu _k , \hat{z}_k- z_k\rangle \nonumber \\&\le \Phi _0(z) + \Vert z_0 - z \Vert ^2+2\sum _{k=0}^{K-1}\Vert \nu _k \Vert ^2+2\sum _{k=0}^{K-1}\langle \nu _k , {\hat{z}}_k - z_k \rangle \nonumber \\&= 2\Vert z_0- z \Vert ^2+ 2\sum _{k=0}^{K-1}\Vert \nu _k \Vert ^2+ 2\sum _{k=0}^{K-1}\langle \nu _k , {\hat{z}}_k - z_k \rangle . \end{aligned}$$

We now take the supremum of this inequality over $z\in {\mathcal {C}}$ and then take a full expectation. As ${\hat{z}}_k$ is ${\mathcal {F}}_k$-measurable, ${\mathbb {E}}[{\mathbb {E}}_k[\cdot ]]={\mathbb {E}}[\cdot ]$, and ${\mathbb {E}}_k \nu _k = 0$, we have ${\mathbb {E}}_k \left[ \langle \nu _k, {\hat{z}}_k - z_k \rangle \right] = 0$. Using this and that $\Phi _{K}(z)\ge 0$ by (16), we arrive at

31 $$\begin{aligned} \tau {\mathbb {E}} \left[ \sup _{z\in {\mathcal {C}}} \sum _{k=0}^{K-1} \Theta _{k+1}(z) \right] \le \sup _{z\in {\mathcal {C}}} \Vert z_0 - z \Vert ^2 + \sum _{k=0}^{K-1} {\mathbb {E}} \Vert \nu _k \Vert ^2. \end{aligned}$$

It remains to estimate the last term $\sum _{k=0}^{K-1} {\mathbb {E}} \Vert \nu _k \Vert ^2$. For this, we use a standard inequality ${\mathbb {E}}\Vert X-{\mathbb {E}} X \Vert ^2\le {\mathbb {E}} \Vert X \Vert ^2$ and Lipschitzness of $F_{i_k}$

32 $$\begin{aligned} \sum _{k=0}^{K-1} {\mathbb {E}} \Vert \nu _k \Vert ^2&= \sum _{k=0}^{K-1} {\mathbb {E}} \tau ^2 \Vert F_{i_k}(z_k) - F_{i_k}(w_{k-1}) - (F(z_k) - F(w_{k-1})) \Vert ^2 \nonumber \\&\le \tau ^2\sum _{k=0}^{K-1} {\mathbb {E}} \Vert F_{i_k}(z_k) -F_{i_k}(w_{k-1}) \Vert ^2 \le \tau ^2 L^2 \sum _{k=0}^{K-1}{\mathbb {E}} \Vert z_k - w_{k-1} \Vert ^2 \nonumber \\&\stackrel{(24)}{\le } \frac{2\tau ^2 L^2(1+\beta )}{\delta \beta }{{\,\mathrm{dist}\,}}(z_0,{\mathcal {Z}}^\star )^2. \end{aligned}$$

Plugging this bound into (31), we obtain

33 $$\begin{aligned}&\tau {\mathbb {E}} \left[ \sup _{z\in {\mathcal {C}}} \sum _{k=0}^{K-1} \Theta _{k+1}(z)\right] \nonumber \\&\quad \le \sup _{z\in {\mathcal {C}}} \Vert z_0 - z \Vert ^2 + \frac{2\tau ^2 L^2(1+\beta )}{\delta \beta }{{\,\mathrm{dist}\,}}(z_0,{\mathcal {Z}}^\star )^2. \end{aligned}$$

Finally, using monotonicity of *F*, followed by Jensen inequality, we deduce

$$\begin{aligned}&\sup _{z\in {\mathcal {C}}} \sum _{k=0}^{K-1} \Theta _{k+1}(z)\nonumber \\&\quad \ge \sup _{z\in {\mathcal {C}}} \sum _{k=1}^{K} \Bigl (\langle F(z),z_k-z\rangle +g(z_k)-g(z)\Bigr )\ge K G_{\mathcal {C}}(z_K^{av}), \end{aligned}$$

which combined with (33) finishes the proof. $$\square 
$$

It is worth mentioning that even though our method is simple and the convergence rate is *O*(1/*k*) as in [5], our complexity result has a worse dependence on *n*, compared to [5]. In particular, our complexity is ${O}(n/\epsilon )$ instead of the ${O}(\sqrt{n}/\epsilon )$ of [5]. This is because our step size has the factor of *p* which is of the order $\frac{1}{n}$ in general and it appears to be tight based on numerical experiments. This seems like the cost of handling a more general problem without bounded domain assumption. We leave it as an open question to derive a method that works under our general assumptions and features favorable complexity guarantees as in [5].

### Convergence rate for strongly monotone case

We show that linear convergence is attained when strong monotonicity is assumed.

#### Theorem 3.3

Let Assumption 1 hold and let *F* be $\mu$-strongly monotone. Let $z^\star$ be the unique solution of (1). Then for the iterates $\{z_k\}$ generated by Algorithm 1 with $\tau = \frac{p}{4\sqrt{2}L}$, it holds that

34 $$\begin{aligned} {\mathbb {E}} \Vert z_k - z^\star \Vert ^2 \le \left( 1- \frac{\mu p}{8\sqrt{2}L} \right) ^k \Vert z_0 - z^\star \Vert ^2. \end{aligned}$$

#### Remark 3.3

We analyzed the case when *F* is strongly monotone, however, the same analysis would go through when *F* is monotone and *g* is strongly convex. One can *transfer* strong convexity of *g* to make *F* strongly monotone.

#### Proof of Theorem 3.3

We start from (8) with $i_k = i$,

$$\begin{aligned}&\Vert z_{k+1} - z \Vert ^2 + 2\tau \langle F(z_{k+1}) - F(w_k), z - z_{k+1} \rangle + 2\tau g(z_{k+1}) - 2\tau g(z)\\&\quad +2\tau \langle F(z_{k+1}), z_{k+1} - z \rangle \le \Vert z_k - z \Vert ^2 + 2\tau \langle F_i(z_k)- F_i(w_{k-1}), z - z_k \rangle \\&\quad + {2\tau \langle F_i(z_k) - F_i(w_{k-1}), z_k - z_{k+1} \rangle } - \Vert z_{k+1} - z_k\Vert ^2 \end{aligned}$$

Setting $z=z^\star$ and using strong monotonicity of *F*,

$$\begin{aligned}&\langle F(z_{k+1}), z_{k+1} - z^\star \rangle + g(z_{k+1})-g(z^\star ) \ge \langle F(z^\star ), z_{k+1} - z^\star \rangle +\mu \Vert z_{k+1}-z^\star \Vert ^2 \\&\quad + g(z_{k+1}) -g(z^\star ) \ge \mu \Vert z_{k+1}-z^\star \Vert ^2. \end{aligned}$$

Hence, we have

$$\begin{aligned}&(1+2\tau \mu ) \Vert z_{k+1} - z^\star \Vert ^2 + 2\tau \langle F(z_{k+1}) - F(w_k), z^\star - z_{k+1} \rangle + \Vert z_{k+1} - z_k\Vert ^2\\&\quad \le \Vert z_k - z^\star \Vert ^2 + 2\tau \langle F_i(z_k)- F_i(w_{k-1}), z^\star - z_k \rangle \\&\quad + {2\tau \langle F_i(z_k) - F_i(w_{k-1}), z_k - z_{k+1} \rangle }. \end{aligned}$$

Then, we continue as in the proof of Theorem 3.1 until we obtain a stronger version of (20) due to the strong monotonicity term

35 $$\begin{aligned}&{\mathbb {E}}_k \biggl [ (1+2\mu \tau )\Vert z_{k+1} - z^\star \Vert ^2 + 2\tau \langle F(z_{k+1}) - F(w_k), z^\star - z_{k+1} \rangle \nonumber \\&\quad + \frac{\beta }{2} \Vert z_k - w_k \Vert ^2 +\frac{1}{2} \Vert z_{k+1} - z_k \Vert ^2 \biggr ] \le \Vert z_{k} - z^\star \Vert ^2 + 2\tau \langle F(z_{k}) - F(w_{k-1}), z^\star - z_{k} \rangle \nonumber \\&\quad + \frac{\beta }{2} \Vert z_{k-1} - w_{k-1} \Vert ^2 +\frac{1}{2} \Vert z_{k} - z_{k-1} \Vert ^2 -\frac{\delta }{2}\Bigl (\Vert z_k-z_{k-1} \Vert ^2 + \beta \Vert z_{k-1}-w_{k-1} \Vert ^2\Bigr ). \end{aligned}$$

Let $$a_{k+1} = \frac{1}{2} \Vert z_{k+1} - z^\star \Vert ^2$$ and

$$\begin{aligned} b_{k+1}&= \frac{1}{2} \Vert z_{k+1} - z^\star \Vert ^2 + 2\tau \langle F(z_{k+1}) - F(w_k), z^\star - z_{k+1} \rangle + \frac{1}{2} \Vert z_{k+1} - z_k\Vert ^2 \\&\qquad + \frac{\beta }{2} \Vert z_k - w_k \Vert ^2. \end{aligned}$$

Note that we have $b_{k+1}+\frac{1}{2}\Vert z_{k+1}-z^\star \Vert ^2=\Phi _{k+1}(z^\star )\ge \frac{1}{2}\Vert z_{k+1}-z^\star \Vert ^2$ by (16), hence $b_{k+1}\ge 0$.

Using the definitions of $a_{k}$ and $b_k$ in (35), it follows that for any $\varepsilon \le \delta$,

36 $$\begin{aligned} {\mathbb {E}}_k\bigl [(1+4\mu \tau ) a_{k+1} + b_{k+1}\bigr ] \le a_{k} + b_{k} - \frac{\varepsilon }{2}\left( \Vert z_{k} - z_{k-1} \Vert ^2 + \beta \Vert z_{k-1} - w_{k-1} \Vert ^2\right) , \end{aligned}$$

Next, we derive

37 $$\begin{aligned}&\text { RHS of } (36)= a_k + b_k - \frac{\varepsilon }{2} \Vert z_k - z_{k-1} \Vert ^2 - \frac{\varepsilon }{2} \beta \Vert z_{k-1} - w_{k-1} \Vert ^2 \end{aligned}$$

38 $$\begin{aligned}&\quad = \left( 1+\frac{\varepsilon }{2} \right) a_k + \left( 1 - \frac{\varepsilon }{2} \right) b_k \nonumber \\&- \frac{\varepsilon }{4} \Vert z_k - z_{k-1} \Vert ^2 - \frac{\varepsilon \beta }{4} \Vert z_{k-1} - w_{k-1} \Vert ^2 \nonumber \\&\quad + \varepsilon \tau \langle F(z_k) - F(w_{k-1}), z^\star - z_k \rangle \le \left( 1+\frac{3\varepsilon }{2}\right) a_k + \left( 1 - \frac{\varepsilon }{2} \right) b_k, \end{aligned}$$

where the last inequality follows from (15) with a shifted index *k*. Then, (36) becomes

39 $$\begin{aligned} {\mathbb {E}}_k\bigl [(1+4\mu \tau ) a_{k+1} + b_{k+1}\bigr ] \le \left( 1+\frac{3\varepsilon }{2}\right) a_k + \left( 1- \frac{\varepsilon }{2} \right) b_k. \end{aligned}$$

Since $\varepsilon \le \delta$ is arbitrary, we can choose $\varepsilon$ such that $1+4\mu \tau > 1+\frac{3\varepsilon }{2}$. For instance, we can set

40 $$\begin{aligned} \varepsilon = \min \left\{ \delta , 2\mu \tau \right\} , \end{aligned}$$

that results in

41 $$\begin{aligned}&{\mathbb {E}}_k\bigl [(1+4\mu \tau ) a_{k+1} + b_{k+1}\bigr ] \le (1+3\mu \tau )a_k + \left( 1 - \frac{\varepsilon }{2} \right) b_k \nonumber \\&\quad = \left( 1 - \frac{\mu \tau }{1+4\mu \tau } \right) (1+4\mu \tau ) a_k + \left( 1 - \frac{\varepsilon }{2} \right) b_k \nonumber \\&\quad \le \left( 1- \min \left\{ \frac{\mu \tau }{1+4\mu \tau }, \frac{\varepsilon }{2} \right\} \right) \bigl ( (1+4\mu \tau )a_k + b_k \bigr ). \end{aligned}$$

Taking a full expectation and using that $\frac{\varepsilon }{2}=\min \{\frac{\delta }{2},\mu \tau \}$ and $b_0=0$, we obtain

$$\begin{aligned} {\mathbb {E}}\bigl [(1+4\mu \tau ) a_{k+1} + b_{k+1}\bigr ]&\le \left( 1- \min \left\{ \frac{\mu \tau }{1+4\mu \tau }, \frac{\delta }{2} \right\} \right) {\mathbb {E}}\bigl [ (1+4\mu \tau )a_k + b_k \bigr ]\\&\le \left( 1- \min \left\{ \frac{\mu \tau }{1+4\mu \tau }, \frac{\delta }{2} \right\} \right) ^{k+1} (1+4\mu \tau )a_0. \end{aligned}$$

Now it only remains to compute the contraction factor. By our choice of $\tau$, we have $\tau L = \frac{p}{4\sqrt{2}} \le \frac{1-\sqrt{1-p}}{2\sqrt{2}} = \frac{\beta }{2\sqrt{2}(1+\beta )}$, and hence,

42 $$\begin{aligned} \delta = \frac{\beta }{1+\beta }-\frac{4\tau ^2 L^2(1+\beta )}{\beta } \ge \frac{\beta }{2(1+\beta )} \ge \frac{1-\sqrt{1-p}}{2} \ge \frac{p}{4}. \end{aligned}$$

From $\mu \le L$ it follows that $4\mu \tau = \frac{\mu p}{\sqrt{2}L}\le \frac{p}{\sqrt{2}}< 1$ and, hence, $\frac{\mu \tau }{1+4\mu \tau }\ge \frac{\mu \tau }{2} =\frac{\mu p}{8\sqrt{2}L}$. Thus, we obtain

$$\begin{aligned}\min \left\{ \frac{\mu \tau }{1+4\mu \tau }, \frac{\delta }{2} \right\} \ge \min \left\{ \frac{\mu p}{8\sqrt{2}L}, \frac{p}{8} \right\} =\frac{\mu p}{8\sqrt{2} L},\end{aligned}$$

which finally implies

$$\begin{aligned} {\mathbb {E}}\Vert z_{k+1} - z^\star \Vert ^2 \le \left( 1-\frac{\mu p}{8\sqrt{2}L} \right) ^{k+1} \Vert z_0 - z^\star \Vert ^2. \end{aligned}$$

$$\square$$

A key characteristic of our result is that strong monotonicity constant is not required in the algorithm as a parameter to obtain the rate. This has been raised as an open question by [2] and a partial answer is studied by [7] (see Table 1). Our result gives a full answer to this question without using strong monotonicity constant in all cases.

We next discuss the dependence of $\mu$ in the convergence rate. Our rate has a dependence of $\frac{1}{\mu }$ compared to $\frac{1}{\mu ^2}$ of non-accelerated methods of [2] and the method of [7]. This difference is important especially when $\mu$ is small. On the other hand, in terms of *n*, our complexity has a worse dependence compared to [5] and accelerated method of [2] as discussed before (see the discussions in Sect. 1.1 and Section 3.2).

### Beyond monotonicity

Lastly, we illustrate that our method has convergence guarantees for a class of non-monotone problems. There exist several relaxations of monotonicity that are used in the literature [10, 17, 22, 24]. Among these, we assume the existence of the solutions to Minty variational inequality given as

43 $$\begin{aligned} \exists {\hat{z}} \in {\mathcal {Z}}:\quad \langle F(z), z - {\hat{z}} \rangle + g(z) - g({\hat{z}}) \ge 0, \quad \forall z\in {\mathcal {Z}}. \end{aligned}$$

Under (43), we can drop the monotonicity assumption and show almost sure subsequential convergence of the iterates of our method. Naturally, in this case one can no longer show sequential convergence as with monotonicity (see Theorem 3.1).

#### Theorem 3.4

Suppose that Assumption 1 (a), (c), (d) and the condition (43) hold. Then almost surely all cluster points of the sequence $\{z_k\}$ generated by Algorithm 1 are in ${\mathcal {Z}}^\star$.

#### Proof

We will proceed as in Theorem 3.1 and [22, Theorem 6]. We note that Lemma 3.2 does not use monotonicity of *F*, thus its result follows in this case. In the inequality

$$\begin{aligned} {\mathbb {E}}_{k} [\Phi _{k+1}(z) +2\tau \Theta _{k+1}(z)] \le \Phi _{k}(z). \end{aligned}$$

we plug in $z=\hat{z}$ for a point satisfying (43).

Then, by (43), we have

$$\begin{aligned} \Theta _{k+1}({\hat{z}})= \langle F(z_{k+1}), z_{k+1} - {\hat{z}} \rangle +g(z_{k+1}) - g({\hat{z}}) \ge 0. \end{aligned}$$

We then argue the same way as in Theorem 3.1 to conclude that almost surely, $\{z_k\}$ is bounded and cluster points of $\{z_k\}$ are in ${\mathcal {Z}}^\star$.

Note that the steps in Theorem 3.1 for showing sequential convergence relies on the choice of *z* as an arbitrary point in ${\mathcal {Z}}^\star$, which is not the case here, therefore, we can only use the arguments from Theorem 3.1 for showing subsequential convergence.$\square$

## Extensions

We illustrate extensions of our results to monotone inclusions and Bregman projections. The proofs for this section are given in the appendix in Section 7.

### Monotone inclusions

We have chosen to focus on monotone VIs in the main part of the paper for being able to derive sublinear rates for the gap function. In this section, we show that our analysis extends directly for solving monotone inclusions. In this case, we are interested in finding *z* such that $0\in (A+F)(z)$, where *A*, *F* are monotone operators and each $F_i$ is Lipschitz with the form $F = \frac{1}{n} \sum _{i=1}^n F_i$. In this case, one changes the prox operator in the algorithm, to resolvent operator of *A* which is defined as $J_{\tau A}(z) = (I+\tau A)^{-1}(z)$. Then, one can use Lemma 3.1 as directly given in [23, Proposition 2.3] to prove an analogous result of Theorem 3.1 for solving monotone inclusions. Moreover, when $A+F$ is strongly monotone, one can prove an analogue of Theorem 3.3. We prove the former result and we note that the latter can be shown by applying the steps in Theorem 3.3 on top of Theorem 4.1, which we do not repeat for brevity.

#### Theorem 4.1

Let $A:{\mathcal {Z}} \rightrightarrows {\mathcal {Z}}$ be maximally monotone and $F:{\mathcal {Z}} \rightarrow {\mathcal {Z}}$ be monotone with $F = \frac{1}{n}\sum _{i=1}^n F_i$, where $F_i$ is *L*-Lipschitz for all *i*. Assume that $(A+F)^{-1}(0)$ is nonempty and let the iterates $\{z_k\}$ be generated by Algorithm 1 with the update for $z_{k+1}$

44 $$\begin{aligned} z_{k+1} = J_{\tau A} (z_k -\tau F(w_k) - \tau (F_{i_k}(z_k) - F_{i_k}(w_{k-1}))). \end{aligned}$$

Then, for $\tau < \frac{1-\sqrt{1-p}}{2L}$, almost surely there exist $z^\star \in (A+F)^{-1}(0)$ such that $z_k \rightarrow z^\star$.

### Bregman distances

We developed our analysis in the Euclidean setting, relying on $\ell _2$-norm for simplicity. However, we can also generalize it to proximal operators involving Bregman distances. In this setting, we have a distance generating function $h:{\mathcal {Z}}\rightarrow {\mathbb {R}}$, which is 1-strongly convex and continuous. We follow the standard convention to assume that subdifferential of *h* admits a continuous selection, which means that there exists a continuous function $\nabla h$ such that $\nabla h(x)\in \partial h(x)$ for all $x\in {{\,\mathrm{dom}\,}}\partial h$. We define the Bregman distance as $D_{h}(z, {\bar{z}}) = h(z) - h({\bar{z}}) - \langle \nabla h({\bar{z}}), z-{\bar{z}} \rangle$. Then, we will change the proximal step 4 of Algorithm 1 with

45 $$\begin{aligned} z_{k+1} = {{\,\mathrm{argmin}\,}}_{z}\Bigl \{ g(z) + \langle F(w_k) + F_{i_k}(z_k) - F_{i_k}(w_{k-1}), z - z_k \rangle + \frac{1}{\tau } D_{h}(z, z_k)\Bigr \}. \end{aligned}$$

We prove an analogue of Lemma 3.2 with Bregman distances from which the convergence rate results will follow.

#### Lemma 4.1

Let Assumption 1 hold and

$$\begin{aligned} \Phi _{k+1}(z) :=D_h(z, z_{k+1}) + \tau \langle F(z_{k+1}) - F(w_k), z- z_{k+1} \rangle&+ \frac{\beta }{4} \Vert z_k - w_k \Vert ^2 \\&+ \frac{1}{2} D_h(z_{k+1}, z_k). \end{aligned}$$

Moreover, suppose $\tau < \frac{1-\sqrt{1-p}}{2L}$, $\beta = \frac{1}{\sqrt{1-p}}-1$, and the iterates $\{z_k\}$ are generated by Algorithm 1 with the update (45) for $z_{k+1}$. Then for any $z\in {{\,\mathrm{dom}\,}}g$,

$$\begin{aligned} {\mathbb {E}}_k[ \Phi _{k+1}(z) + \tau \Theta _{k+1}(z)] \le \Phi _k(z). \end{aligned}$$

## Numerical verification

In this section, we include preliminary experimental results for our algorithm. We would like to note that these results are mainly for verifying our theoretical results and are not intended to serve as complete benchmarks. We suspect that for an extensive practical comparison, some practical enhancements of our method similar to proximal-point acceleration from [2] or restarting from [7] may be useful. We leave such investigations for future work.

First, we apply our method to the *unconstrained* bilinear problem. It was shown in [7] that this simple problem is particularly challenging for stochastic methods, due to unboundedness of the domain, where the standard methods, such as stochastic extragradient method [19], diverges. Our assumptions are general enough to cover this case and we now verify in practice that our method indeed converges for this problem by setting $d=n=100$ and generating $A_i\in {\mathbb {R}}^{d\times d}$ randomly with distribution ${\mathcal {N}}(0, 1)$

46 $$\begin{aligned} \min _{x\in {\mathbb {R}}^{d}}\max _{y\in {\mathbb {R}}^{d}}\frac{1}{n} \sum _{i=1}^n \langle A_ix, y \rangle . \end{aligned}$$

**Fig. 1:**
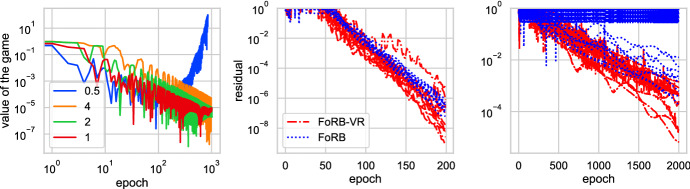
Left: bilinear problem. Middle: Constrained minimization with data generated by normal distribution. Right: Constrained minimization with data generated by uniform distribution

For this experiment, we test the tightness of our step size rule by progressively increasing it. Recall that our step size is $\tau = \frac{p}{cL}$, where $c=4$ is suggested in our analysis, see Corollary 3.1. We try the values of $c = \{ 0.5, 1, 2, 4\}$ and observe that for $c=0.5$ the algorithm diverges, see Fig. 1(left). The message of this experiment is that even though slightly higher step sizes than what is allowed in our theory might work, it is not possible to significantly increase it.

The second problem we consider is constrained minimization, which is an instance where the dual domain is not necessarily bounded. We want to solve

$$\begin{aligned} \min _{x\in C} f(x) \quad \text {s.t.} \quad h_i(x) \le 0, \quad i=1,\dots , m, \end{aligned}$$

where $f(x) = \frac{1}{2} \Vert x-u \Vert ^2$ for some $u \in {\mathcal {Z}}$ and $h_i(x) = \Vert A_i x - b_i\Vert ^2 - \delta _i$ for $A_i \in {\mathbb {R}}^{d\times d}$, $b_i \in {\mathbb {R}}^d$, $\delta _i \in \mathbb {R_{++}}$, and *C* is a unit ball. In other words, we want to find a projection of *u* onto the intersection given by *C* and the constraint inequalities $\{x:h_i(x)\le 0\}$.

Introducing Lagrange multipliers $y_i$ for each constraint, we obtain (see Section 7.3 for further details)

$$\begin{aligned} \min _{x\in {\mathbb {R}}^d}\max _{y \in {\mathbb {R}}^m_+} f(x) + \sum _{i=1}^m y_i h_i(x). \end{aligned}$$

As the Lipschitz constant in this problem does not admit a closed-form expression, we first estimate the Lipschitz constant by finding an *L* such that deterministic method converges. Next, we note that even though we analyzed the algorithm with a single step size $\tau$ for both primal and dual variables *x*, *y*, one can use different step sizes for primal and dual variables (see [22, Section 4.1]). Therefore, we tuned the scaling of primal and dual step sizes for both methods with one random instance and we used the same scaling for all tests for both methods.

We set $p=1/m$. Every iteration, the deterministic method needs to go through all *m* constraints to compute $\sum _{i=1}^my_i\nabla h_i(x)$, whereas our method computes $\nabla h_i(x)$ for only one *i*. First setup is with $m=400$, $d=100$, and the data is generated with the normal distribution ${\mathcal {N}}(0, 1)$. Second setup is with $m=400$, $d=50$, and the data is generated with the uniform distribution ${\mathcal {U}}(-1, 1)$. We ran both setups with 10 different instances of randomly generated data and plotted all results, see Fig. 1. We observe that in one instance, the tuned scaling diverges for deterministic method, whereas our method with the same tuning converged in all cases.

## Conclusion

In this work, we proposed a variance reduced algorithm for solving monotone VIs without assuming bilinearity, strong monotonicity, cocoercivity or boundedness. Even though our method is the first to converge under the same set of assumptions as deterministic methods, a drawback of our approach is the lack of complexity improvements.

In particular, previous approach of [5] showed complexity improvements for bilinear games, while needing more assumptions than deterministic methods to converge. Thus, an important open problem is to obtain a method that *i*) converges under the minimal set of assumptions as our algorithm, *ii*) features improved complexity guarantees compared to deterministic methods, while solving structured problems such as bilinear games such as [5] to obtain the best of both worlds.

## 7. Appendix

### 7.1 Proofs for Sect. 3

#### Proof of Lemma 3.1

By using the definition of proximal operator and convexity of *g*, we have for all $x\in {\mathcal {Z}}$

47 $$\begin{aligned} g(x)&\ge g(x_2) + \langle x_1 - U_1 - (V_1 - U_0) - x_2, x-x_2 \rangle \nonumber \\&= g(x_2) + \langle x_1 - x_2, x - x_2 \rangle - \langle U_1, x - x_2 \rangle - \langle V_1 - U_0, x - x_2 \rangle . \end{aligned}$$

Since $2\langle a, b \rangle = \Vert a \Vert ^2 + \Vert b\Vert ^2 - \Vert a-b\Vert ^2$, $\forall a,b$, it follows that

$$\begin{aligned} 2 \langle x_1 - x_2, x - x_2 \rangle = \Vert x_1 - x_2 \Vert ^2 + \Vert x - x_2 \Vert ^2 - \Vert x - x_1 \Vert ^2. \end{aligned}$$

Simple rearrangements give

$$\begin{aligned} -\langle U_1, x-x_2 \rangle = \langle V_2 - U_1, x - x_2 \rangle - \langle V_2, x - x_2 \rangle \end{aligned}$$

and

$$\begin{aligned} -\langle V_1 - U_0, x - x_2 \rangle = -\langle V_1 - U_0, x - x_1 \rangle -\langle V_1 - U_0, x_1 - x_2 \rangle . \end{aligned}$$

Using the last three equalities in (47) completes the result. $\square$

### 7.2 Proofs for Sect. 4

We first need a generalized version of Lemma 3.1. In fact, this is the exact form proven in [23], therefore we do not provide its proof.

#### Lemma 7.1

[23, Proposition 2.3] Let $A:{\mathcal {Z}} \rightrightarrows {\mathcal {Z}}$ be maximally monotone and let $x_1, U_{0}, U_1$, $V_1\in {\mathcal {Z}}$ be arbitrary points. Define $x_{2}$ as

48 $$\begin{aligned} x_{2} = J_{A}(x_1-U_1 - (V_1 - U_{0})). \end{aligned}$$

Then for all $x\in {\mathcal {Z}}$, $V_2\in {\mathcal {Z}}$, and $U\in -A(x)$, we have

49 $$\begin{aligned}&\Vert x_{2}-x \Vert ^2 + 2\langle V_{2}-U_1, x-x_{2}\rangle + 2\langle V_2 - U, x_2 - x \rangle \nonumber \\&\quad \le \Vert x_{1}-x \Vert ^2 + 2\langle V_{1}-U_{0}, x-x_{1}\rangle + 2\langle V_1-U_{0},x_1-x_{2}\rangle - \Vert x_{1}-x_2 \Vert ^2 . \end{aligned}$$

#### 7.2.1 Proof of Theorem 4.1

##### Proof

We will start similar to Lemma 3.2. After setting $U_0 =\tau F_i(w_{k-1})$, $U_1=\tau F(w_k)$, $V_1 =\tau F_i(z_k)$, $V_2 =\tau F(z_{k+1})$, $x_1 = z_k$, $x_2 = z_{k+1}$ with $i_k = i$ and plugging into Lemma 7.1, we have

50 $$\begin{aligned}&\Vert z_{k+1} - z \Vert ^2 + 2\tau \langle F(z_{k+1}) - F(w_k), z - z_{k+1} \rangle \le \Vert z_k - z\Vert ^2 \nonumber \\&\quad + 2\tau \langle F_i(z_k)- F_i(w_{k-1}), z-z_k \rangle \nonumber \\&\quad + {2\tau \langle F_i(z_k) - F_i(w_{k-1}), z_k - z_{k+1} \rangle } - \Vert z_{k+1} - z_k\Vert ^2 \nonumber \\&\quad - 2\tau \langle F(z_{k+1}) - F(z), z_{k+1} - z \rangle . \end{aligned}$$

We use monotonicity for the last term and get

51 $$\begin{aligned}&\Vert z_{k+1} - z \Vert ^2 + 2\tau \langle F(z_{k+1}) - F(w_k), z - z_{k+1} \rangle \le \Vert z_k - z\Vert ^2 - \Vert z_{k+1} - z_k\Vert ^2\nonumber \\&\quad + 2\tau \langle F_i(z_k)- F_i(w_{k-1}), z-z_k \rangle + {2\tau \langle F_i(z_k) - F_i(w_{k-1}), z_k - z_{k+1} \rangle }. \end{aligned}$$

The rest of Lemma 3.2 follows in this case the same way with the lack of the terms with $\Theta _{k+1}(z)$. Then, similar arguments as in Theorem 3.1 with the changes of *i*) not having $\Theta _{k+1}(z)$, *ii*) using the definition of resolvent instead of proximal operator to show cluster points are solutions, will give the result (see also [23, Theorem 2.5]). $\square$

We now present a version of Lemma 3.1 with the proximal operator using Bregman distance.

##### Lemma 7.2

Let $g:{\mathcal {Z}}\rightarrow {\mathbb {R}}\cup \{+\infty \}$ be proper lower semicontinuous convex and let $x_1, U_{0}, U_1$, $V_1\in {\mathcal {Z}}$ be arbitrary points. Define $x_{2}$ as

52 $$\begin{aligned} x_{2} = {{\,\mathrm{argmin}\,}}_{z\in {\mathcal {Z}}}\Bigl \{ g(z) + \langle U_1 + (V_1 - U_{0}), z-x_1 \rangle + D_h(z, x_1)\Bigr \}. \end{aligned}$$

Then, for all $x\in {\mathcal {Z}}$, $V_2 \in {\mathcal {Z}}$ we have

53 $$\begin{aligned}&D_h(x, x_2) + \langle V_{2}-U_1, z-x_{2}\rangle +\langle V_{2}, x_{2}-x\rangle + g(x_2) - g(x) \nonumber \\&\quad \le D_h(x, x_1) + \langle V_{1}-U_{0}, x-x_{1}\rangle + \langle V_1-U_{0},x_1-x_{2}\rangle - D_h(x_2, x_1) . \end{aligned}$$

##### Proof

By the definition of $x_2$, it follows from [35, Property 1] that

$$\begin{aligned} g(x) \ge g(x_2) - \langle U_1 + V_1 - U_0, x-x_2 \rangle - D_h(x, x_1) + D_h(x, x_2) + D_h(x_2, x_1). \end{aligned}$$

For the bilinear term, we argue the same as Lemma 3.1. $\square$

#### 7.2.2 Proof of Lemma 4.1

##### Proof

We will follow the proof of Lemma 3.2 with suitable changes for Bregman distances.

First, set $U_0 =\tau F_i(w_{k-1})$, $U_1=\tau F(w_k)$, $V_1 =\tau F_i(z_k)$, $V_2 =\tau F(z_{k+1})$, $x_1 = z_k$, then $x_2 = z_{k+1}$ with $i_k=i$ and we plug these into (53) to get

$$\begin{aligned}&D_h(z, z_{k+1}) + \tau \langle F(z_{k+1}) - F(w_k), z - z_{k+1} \rangle + \tau (\langle F(z_{k+1}), z_{k+1} - z \rangle \\&\quad + g(z_{k+1}) - g(z) ) \le D_h(z, z_k) + \tau \langle F_i(z_k)- F_i(w_{k-1}), z-z_k \rangle \\&\quad +{\tau \langle F_i(z_k) - F_i(w_{k-1}), z_k - z_{k+1} \rangle } - D_h(z_{k+1}, z_k). \end{aligned}$$

First, note that by Lipschitzness of $F_i$, Cauchy-Schwarz, Young’s inequalities, and since $\frac{1}{2} \Vert z_k - z_{k-1} \Vert ^2 \le D_h(z_k, z_{k-1})$,

54 $$\begin{aligned}&\tau \langle F_i(z_k) - F_i(w_{k-1}), z_k - z_{k+1} \rangle \nonumber \\&\quad \le \tau ^2L^2 \Vert z_k - w_{k-1}\Vert ^2 + \frac{1}{4} \Vert z_k - z_{k+1} \Vert ^2 \nonumber \\&\quad \le \tau ^2L^2 \Vert z_k - w_{k-1}\Vert ^2 + \frac{1}{2} D_h(z_{k+1}, z_k) \end{aligned}$$

Thus, it follows that

55 $$\begin{aligned} D_h(z, z_{k+1})&+ \tau \langle F(z_{k+1}) - F(w_k), z - z_{k+1} \rangle + \frac{1}{2} D_h(z_{k+1}, z_k)+ \tau \Theta _{k+1}(z) \nonumber \\ \le D_h(z, z_k)&+ \tau \langle F_i(z_k)- F_i(w_{k-1}), z-z_k \rangle +\tau ^2L^2 \Vert z_k - w_{k-1}\Vert ^2. \end{aligned}$$

Taking expectation conditioning on the knowledge of $z_k, w_{k-1}$ and using that ${\mathbb {E}}_k F_i (z_k) = F(z_k)$, ${\mathbb {E}}_k F_i(w_{k-1}) = F(w_{k-1})$, we obtain

56 $$\begin{aligned}&{\mathbb {E}}_k D_h(z, z_{k+1}) + \tau {\mathbb {E}}_k \langle F(z_{k+1}) -F(w_k), z - z_{k+1} \rangle \nonumber \\&\quad +\frac{1}{2} {\mathbb {E}}_k D_h(z_{k+1}, z_k) +\tau {\mathbb {E}}_k \Theta _{k+1}(z) \nonumber \\&\quad \le D_h(z, z_k) + \tau \langle F(z_k) - F(w_{k-1}), z-z_k \rangle + \tau ^2L^2 \Vert z_{k} - w_{k-1} \Vert ^2. \end{aligned}$$

Adding

57 $$\begin{aligned} \frac{\beta }{4}{\mathbb {E}}_{k} \Vert z_k - w_k \Vert ^2 = \frac{\beta (1-p)}{4} \Vert z_k - w_{k-1} \Vert ^2, \end{aligned}$$

which follows by the definition of $w_k$, to (56), we obtain

58 $$\begin{aligned}&{\mathbb {E}}_k [\Phi _{k+1}(z) +\Theta _{k+1}(z)] \le \Phi _{k}(z) \nonumber \\&\quad +\Big (\tau ^2L^2+\frac{\beta (1-p)}{4} \Big )\Vert z_{k} - w_{k-1} \Vert ^2-\frac{1}{2}D_h(z_k, z_{k-1}) - \frac{\beta }{4}\Vert z_{k-1}-w_{k-1} \Vert ^2. \end{aligned}$$

To show that the last line is nonpositive, we use (14), Young’s inequality as in Lemma 3.2 and $\frac{1}{2} \Vert z_k - z_{k-1} \Vert ^2 \le D_h(z_k, z_{k-1})$.

Nonnegativity of $\Phi _k$ follows as in Theorem 3.1 after using $\frac{1}{2} \Vert z_k - z_{k-1} \Vert ^2 \le D_h(z_k, z_{k-1})$. $\square$

### 7.3 Experiment details

Only for this section, we will use superscripts for iterates rather than subscripts that we have used up to now. Recall that our problem is

$$\begin{aligned} \min _{x\in {\mathbb {R}}^d}\max _{y \in {\mathbb {R}}^m_+} f(x) + \sum _{i=1}^m y_i h_i(x). \end{aligned}$$

This problem is equivalent to the following variational inequality

$$\begin{aligned} \langle F(z^*), z-z^*\rangle \ge 0 \quad \forall z\in C\times {\mathbb {R}}^m_+, \end{aligned}$$

where

$$\begin{aligned} z = (x,y),\quad F(z) = \left( {\begin{array}{c}\nabla f(x) + \sum _{i=1}^my_i\nabla h_i(x)\\ -h(x)\end{array}}\right) =\left( {\begin{array}{c}F^{(1)}(z)\\ F^{(2)}(z)\end{array}}\right) \end{aligned}$$

The notation reads: $h(x) = (h_1(x),\dots , h_m(x))$ and $\nabla h(x) = (\nabla h_1(x),\dots , \nabla h_m(x))$. Let us note $h:{\mathbb {R}}^d \rightarrow {\mathbb {R}}^m$, $\nabla h(x)\in {\mathbb {R}}^{m\times d}$. We note that the residual in *y*-axes of Fig. 1 is computed as $\Vert x_t - {{\,\mathrm{prox}\,}}_g(x_t - F(x_t)) \Vert$.

We split *F* as follows

$$\begin{aligned} F(z)=\frac{1}{m}\sum _{i=1}^mF_i(z) \quad \text {with} \quad F_i(z)=\left( {\begin{array}{c} f(x)+ m y_i\nabla h_i(x)\\ -m h_i(x) {\mathbf {e}}_i\end{array}}\right) =\left( {\begin{array}{c}F_i^{(1)}(z)\\ F_i^{(2)}(z)\end{array}}\right) , \end{aligned}$$

where $({\mathbf {e}}_i)_{i=1}^m$ is a standard basis in ${\mathbb {R}}^m$.

Hence, Algorithm 1, with different step sizes for primal and dual, will be

59 $$\begin{aligned} \begin{aligned} x^{k+1} = P_C\big (x^k -\tau F^{(1)}(u^k, v^k) - \tau (F_i^{(1)}(x^k,y^k) - F_i^{(1)}(u^{k-1}, v^{k-1}))\big ) \\ y^{k+1} = P_{{\mathbb {R}}_{m}^+}(y^k -\sigma F^{(2)}(u^k, v^k) - \sigma (F_i^{(2)}(x^k,y^k) - F_i^{(2)}(u^{k-1}, v^{k-1}))\big )\\ (u^{k+1},v^{k+1}) = {\left\{ \begin{array}{ll} (x^{k+1},y^{k+1})&{} \text {with probability }\ p\\ (u^k,v^k)&{} \text {with probability }\ 1-p \end{array}\right. } \end{aligned} \end{aligned}$$

## References

[1] Alacaoglu, A., Fercoq, O., Cevher, V. On the convergence of stochastic primal-dual hybrid gradient. arXiv preprint arXiv:1911.00799, (2019)

[2] Balamurugan, P., and Bach. F. Stochastic variance reduction methods for saddle-point problems. In Advances in Neural Information Processing Systems, pages 1416–1424, (2016)

[3] Bertsekas DP (2011). Incremental proximal methods for large scale convex optimization. Math. Prog..

[4] Boţ R. I., Mertikopoulos, P., Staudigl, M., and Vuong, P. T. Forward-backward-forward methods with variance reduction for stochastic variational inequalities. arXiv preprint arXiv:1902.03355, (2019)

[5] Carmon, Y., Jin, Y., Sidford, A., and Tian. K. Variance reduction for matrix games. In Advances in Neural Information Processing Systems, pages 11377–11388, (2019)

[6] Chambolle A, Pock T (2011). A first-order primal-dual algorithm for convex problems with applications to imaging. J. Math. Imag. Vis..

[7] Chavdarova, T., Gidel G., Fleuret., F and Lacoste-Julien., S. Reducing noise in GAN training with variance reduced extragradient. In Advances in Neural Information Processing Systems, pages 391–401, (2019)

[8] Combettes PL, Pesquet J-C (2015). Stochastic quasi-Fejér block-coordinate fixed point iterations with random sweeping. SIAM J. Optim..

[9] Cui., S and Shanbhag., UV. On the analysis of variance-reduced and randomized projection variants of single projection schemes for monotone stochastic variational inequality problems. arXiv preprint arXiv:1904.11076, (2019)

[10] Dang CD, Lan G (2015). On the convergence properties of non-euclidean extragradient methods for variational inequalities with generalized monotone operators. Comput. Opt. Appl..

[11] Daskalakis. C., and Panageas. I. The limit points of (optimistic) gradient descent in min-max optimization. In Advances in Neural Information Processing Systems, pages 9236–9246, (2018)

[12] Daskalakis, C., Ilyas, A., Syrgkanis, V., and Zeng, H. Training GANs with optimism. In International Conference on Learning Representations, (2018)

[13] Defazio, A., Bach, F., and Lacoste-Julien, S. Saga: A fast incremental gradient method with support for non-strongly convex composite objectives. In Advances in Neural Information Processing Systems, pages 1646–1654, (2014)

[14] Esser E, Zhang X, Chan TF (2010). A general framework for a class of first order primal-dual algorithms for convex optimization in imaging science. SIAM J. Imag. Sci..

[15] Hamedani, E. Y., and Aybat, N. S. A primal-dual algorithm for general convex-concave saddle point problems. arXiv:1803.01401, (2018)

[16] Hofmann, T., Lucchi, A., Lacoste-Julien, S., and McWilliams, B. Variance reduced stochastic gradient descent with neighbors. In Advances in Neural Information Processing Systems, pages 2305–2313, (2015)

[17] Iusem AN, Jofré A, Oliveira RI, Thompson P (2017). Extragradient method with variance reduction for stochastic variational inequalities. SIAM J. Opt..

[18] Johnson, R., and Zhang. T. Accelerating stochastic gradient descent using predictive variance reduction. In Advances in Neural Information Processing Systems, pages 315–323, (2013)

[19] Juditsky A, Nemirovski A, Tauvel C (2011). Solving variational inequalities with stochastic mirror-prox algorithm. Stoch. Syst..

[20] Korpelevich G (1976). The extragradient method for finding saddle points and other problems. Ekon. Mat. Metody.

[21] Kovalev, D., Horvath, S., and Richtarik. P. Don’t jump through hoops and remove those loops: SVRG and Katyusha are better without the outer loop. In Proceedings of the 31st International Conference on Algorithmic Learning Theory, pages 451–467, (2020)

[22] Malitsky Y (2019). Golden ratio algorithms for variational inequalities. Math. Prog..

[23] Malitsky Y, Tam MK (2020). A forward-backward splitting method for monotone inclusions without cocoercivity. SIAM J. Optim..

[24] Mertikopoulos, P., Lecouat, B., Zenati, H., Foo, C. S., Chandrasekhar, V., and Piliouras. G. Optimistic mirror descent in saddle-point problems: Going the extra(-gradient) mile. In International Conference on Learning Representations, (2019)

[25] Mishchenko, K., Kovalev, D., Shulgin, E., Richtárik, P., and Malitsky. Y.: Revisiting stochastic extragradient. In The 23rd International Conference on Artificial Intelligence and Statistics, pages 4573–4582. PMLR, (2020)

[26] Mokhtari, A., Ozdaglar, A., and Pattathil. S.: A unified analysis of extra-gradient and optimistic gradient methods for saddle point problems: Proximal point approach. In International Conference on Artificial Intelligence and Statistics, pages 1497–1507. PMLR, (2020)

[27] Nemirovski A (2004). Prox-method with rate of convergence $$O(1/t)$$ for variational inequalities with lipschitz continuous monotone operators and smooth convex-concave saddle point problems. SIAM J. Opt..

[28] Nemirovski A, Juditsky A, Lan G, Shapiro A (2009). Robust stochastic approximation approach to stochastic programming. SIAM J. Opt..

[29] Nesterov Y (2007). Dual extrapolation and its applications to solving variational inequalities and related problems. Math. Prog..

[30] Popov LD (1980). A modification of the Arrow-Hurwicz method for search of saddle points. Math. Notes Acad. Sci. USSR.

[31] Rakhlin, S., and Sridharan. K, Optimization, learning, and games with predictable sequences. In Advances in Neural Information Processing Systems, pages 3066–3074, (2013)

[32] Robbins, H., and Siegmund. D.: A convergence theorem for non negative almost supermartingales and some applications. In Optimizing methods in statistics, pages 233–257. Elsevier, (1971)

[33] Shalev-Shwartz, S., and Zhang. T: Stochastic dual coordinate ascent methods for regularized loss minimization. J. Mach. Learn. Res., 14(Feb):567–599, (2013)

[34] Tseng P (2000). A modified forward-backward splitting method for maximal monotone mappings. SIAM J. Control Opt..

[35] Tseng. P.: On accelerated proximal gradient methods for convex-concave optimization. submitted to SIAM J. Opt., 1, (2008)

